# Glomalin contributed more to carbon, nutrients in deeper soils, and differently associated with climates and soil properties in vertical profiles

**DOI:** 10.1038/s41598-017-12731-7

**Published:** 2017-10-11

**Authors:** Wenjie Wang, Zhaoliang Zhong, Qiong Wang, Humei Wang, Yujie Fu, Xingyuan He

**Affiliations:** 1 Key laboratory of Forest Plant Ecology, Northeast Forestry University, Harbin, 150040 P.R. China; 2 Urban forest and urban wetland group, Northeast Institute of Geography and Agroecology, CAS, Changchun, 130102 P.R. China

## Abstract

Despite vital importance in soil conditioning and a proxy for arbuscular mycorrhizal (AMF), glomalin-related soil protein (GRSP) contribution to soil carbon and nutrients at vertical soil profiles and underlying mechanism were not well-defined yet. Thus, 360 soil samples were collected from 72 farmland 1-m soil profiles in northeastern China, and soil physiochemical properties, nutrients, glomalin characteristics, local climates were determined. Linear decreases of glomalin amounts were observed from the top to deep soils, and glomalin/SOC (glomalin ratio to total SOC) in the 80–100 cm soil (EEG, easily-extracted GRSP, 2.2%; TG, total GRSP, 19%) was 1.34–1.5-fold higher than did in the 0–20 cm soil. Different statistical analyses crosschecked that the lower pH and higher SOC usually accompanied with the higher EEG and TG, while EEG was more sensitive to climates; Moreover, glomalin was more physiochemical-regulated in the deep soils, but more nutrient-regulation was found in the surface soils. Structure Equation Model showed that soil depths and climates indirectly affected TG and EEG features through soil properties, except significant direct effects on EEG. In future, glomalin assessment should fully consider these for identifying the AMF importance in the whole 1-m profile, and our findings also favor degrade soil improvement from glomalin rehabilitation.

## Introduction

Glomalin-related soil protein (GRSP) is a glycoprotein produced by the hyphae of arbuscular mycorrhizal fungi (AMF) that contains large contents of metal ions^1,2^. GRSP amount received increasing attention over the past few years for the anti-erosion ability of GRSP in soil aggregate formation^3–5^ and the physical networking function of AM hyphae^6^, loading capacity for heavy metals^2^, maintaining soil fertility^7^, increasing soil C capture^8–11^ and enhancing the availability of polycyclic aromatic hydrocarbons in soil^12^. Many studies proved that GRSP has a complex composition of asparagine-linked carbohydrate chains^13,14^ and it is a mixture of organic matter, amino acids and carbohydrates along with some aliphatic methines, methylenes^3^ and possibly some auto-fluorescent compounds tightly bound with metal ions^15,16^. However, limited information is available regarding glomalin variation at vertical soil profiles and underlying mechanism related soil properties and climatic variations, and this information will benefit the local degraded soil improvements and exact evaluations of AMF-importance^14,16,17^.

Shallow-soil-sampling is often justified by assuming that deeper soil horizons are stable and will not change over time, although some medium- and long-term studies do not support this assumption^18^. In a shallow 40 cm soil profile, soil glomalin exhibits obvious vertical decreasing pattern^19^, and supply of fresh plant-derived carbon to the subsoil (0.6–0.8 m depth) stimulated the microbial mineralization of 2,567 plus minus 226-year-old carbon^20^. The fraction of soil organic carbon (SOC) that may be attributed from glomalin, glomalin/SOC, may be taken as a probe of either enhanced stability or preferential production of glomalin^21^. Given that glomalin/SOC or glomalin/nutrients ratio is higher in the deep soils, glomalin became more important in the deep soils than did in the surface soils, owing to their possible higher contribution to soil C sequestration and nutrient storage; And the definition of this hypothesis needs a soil sampling at the deep soil profiles. The total AMF infection level and glomalin (easily-extracted GRSP, EEG; and total GRSP, TG) were positively correlated with soil edaphic factors, indicating that AM fungal infections and glomalin may be useful to monitor desertification and degraded soil improvement^22^. Within the top 100 cm of farmland soil, AMF declined with increasing soil depth, however, AMF diversity was not different by soil depth^23^. As a proxy of AMF biomass, the vertical glomalin distribution in a deep soil profile and possible associations with soil properties and climates at sampling sites did not fully be analyzed to date. Thus, a clear understanding of differences between the surface and deep soils in glomalin characteristics and possible underlying mechanism, e.g., associations with soil properties and sampling site climatic conditions, is essential for the scientific assessments of glomalin and AMF importance for farmland productivity in NE China.

Technological advances have enabled a new class of multivariate models for ecology, with the potential now to specify a statistical model for abundances jointly across many taxa, to simultaneously explore interactions across taxa and the response of abundance to environmental variables^24^. Structural equation modeling (SEM) is a powerful, multivariate technique found increasingly in scientific investigations to test and evaluate multivariate causal relationships^25^, and powerful in soil ecology from patterns to causalunderstanding^26^. Pearson correlation and stepwise regression are common-used methods for finding complex associations and possible casual relations^27–30^. Canonical ordinations are invaluable tools for modeling communities through environmental predictors, and variation partitioning can then be used to test and determine the likelihood of these sets of predictors in explaining patterns in community structure^31^, is also powerful in identifying co-occurring species on translocation success in *Iris atrofusca* ^32^. Ecologists and evolutionary biologists rely on an increasingly sophisticated set of statistical tools to describe complex natural systems^33^, and a large field dataset together with multiple statistical tools is possibly cross-checking the reliability of the results and also underlying mechanism clarifications^34,35^.

In this paper, we hypothesized that both GRSP amounts and their contribution to SOC and nutrients were soil depth-dependent, and these glomalin features could be strongly regulated by soil properties and climatic differences. Three questions will be answered in this paper, i.e., 1) What’re the differences of glomalin at a 1-m soil profile [including EEG, TG, EEG/TG and their contribution to SOC and nutrients]? 2) What’s the differences of various soil nutrients (SOC, nitrogen [N], available N [AN], phosphorus [P], available P [AP], potassium [K] and available K [AK]) and soil physiochemical properties (pH, electrical conductivity [EC] and soil water) at a 1-m soil profile? 3) which factors of soil nutrients, physiochemical properties and climatic conditions at sampling sites are responsible for the glomalin variations, and how large differences existed in the deep and surface soils? The answers to abovementioned questions may support possible scientific evaluation of glomalin importance in soil improvement and possible measures for degraded soil rehabilitation from the viewpoint of glomalin recovery.

## Results

### Glomalin vertical variations: average and deviation

Two components of glomalin (EEG and TG) linearly decreased from the top soil to the deep soil (*p* < 0.001), and peak values in the surface soil were 0.742 g kg^−1^ for EEG, 6.041 g kg^−1^ for TG (Table 1). Their ratio to total SOC linearly increased with soil depth, showing the much higher glomalin contribution to SOC in the deep soils. For example, in the deep 40–100 cm soils, 1.11–1.31 fold higher glomalin (EEG and TG)/SOC ratios were found compared with those in the surface 0–40 cm soils. Similarly, EEG/N, TG/N, EEG/P, TG/P showed linear increases with the soil depth. The deviations of glomalin at soil profile increasing with the soil depth was generally observed. For example, in the case of Coefficient of Variation (CV = standard error/average), linear increases were found in CVs of EEG, TG, EEG/SOC, TG/SOC, EEG/N as well as EEG/TG (Table 1). In the case of data deviations (SE), significant increases were found in EEG/SOC, TG/SOC, EEG/N, TG/N, while the linear changes were not statistically significant in EEG/P, TG/ P (data not shown here).

**Table 1:** Differences in EEG and TG amounts at different soil depths. Different lowercases in the same column at each group indicates the differences between soil depths, statistically significant at p < 0.05.

Depth cm	EEG	TG	EEG/SOC	TG/SOC	EEG/N	TG/N	EEG/P	TG/P	EEG/TG
Average of pooled values									
0–20	0.745d	5.80d	0.017a	0.13a	0.022a	0.16ab	0.013a	0.090a	0.14a
20–40	0.563c	4.17c	0.019a	0.13a	0.019a	0.14a	0.010a	0.071a	0.16a
40–60	0.413b	3.50bc	0.018a	0.15ab	0.018a	0.14a	0.013a	0.13ab	0.20a
60–80	0.328ab	3.07ab	0.020a	0.17bc	0.023a	0.19b	0.010a	0.10ab	0.18a
80–100	0.290a	2.40a	0.022a	0.19c	0.024a	0.19b	0.043b	0.25b	0.21a
Linear changes	**y = −0.006x + 0.75**	**y = −0.040x + 5.8**	**y = 7E-05 + 0.02**	**y = 0.001x + 0.11**	y = 5E-05x + 0.02	**y = 0.001x + 0.14**	**y = 0.0003x + 0.003**	**y = 0.002x + 0.04**	y = 0.001x + 0.140
R^2^	**0.230**	**0.228**	**0.02**	**0.06**	0.01	**0.03**	**0.013**	**0.012**	0.009
p-level	**0.000**	**0.000**	**0.011**	**0.000**	0.131	**0.003**	**0.032**	**0.037**	>0.05
CV% (coefficient of variation)									
0–20	0.36	0.37	0.42	0.35	0.51	0.41	2.27	1.92	0.40
20–40	0.53	0.51	0.55	0.46	0.66	0.62	0.77	0.81	0.59
40–60	0.80	0.61	0.67	0.53	0.83	0.51	1.36	2.01	1.35
60–80	0.94	0.68	1.00	0.56	0.97	0.64	1.47	1.42	1.47
80–100	0.98	0.73	0.85	0.69	0.86	0.70	3.77	3.74	1.43

Note: EEG/SOC, relative ratio of the carbon in EEG to total SOC; TG/SOC, relative ratio of the carbon in TG to total SOC; EEG/N, relative ratio of the nitrogen in EEG to total soil N; TG/N, relative ratio of the nitrogen in TG to total soil N; EEG/P, relative ratio of the phosphorus in EEG to total soil P; TG/P, relative ratio of the phosphorus in TG to total soil P; EEG/TG, relative ratio of the EEG to TG.

### Soil property vertical variations

Pooled data regression analysis showed that soil physiochemical properties and soil nutrients showed linearly changes at the vertical profile (Table 2). Soil bulk density and pH showed linear increases with the soil depth, while all others (soil water, EC, SOC, N, AN, P, AP, K and AK) showed linear decreases with the soil depth (Table 2).

**Table 2:** Variations in soil fertility properties at different soil depths (MANOVA) in average and deviation.

Depth cm	Bulk density (g cm^−3^)	Soil water (%)	pH	EC (μS cm^−1^)	SOC (g kg^−1^)	N (g kg^−1^)	AN (mg kg^−1^)	P (g kg^−1^)	AP (mg kg^−1^)	K (g kg^−1^)	AK (mg kg^−1^)
0–20	1.42a	12.52a	7.82a	160.30b	17.46e	1.42d	108.0c	0.47d	8.37b	44.3a	82.67b
20–40	1.45ab	14.34b	7.95a	107.25a	12.7d	1.28c	81.3b	0.36c	6.00a	52.1b	62.97a
40–60	1.46bc	12.69a	8.23b	95.31a	8.86c	0.95b	65.3b	0.28b	5.47a	45.0a	61.3a
60–80	1.50c	11.36a	8.23b	98.23a	7.17b	0.63a	28.4a	0.24ab	5.59a	51.6b	49.3a
80–100	1.50c	11.28a	8.28b	93.77a	5.22a	0.51a	34.1a	0.20a	5.56a	60.2c	53.0a
Linear changes	**y = 0.001x + 1.4**	**y = −0.03x + 13.9**	**y = 0.006x + 7.8**	**y = −0.71 + 146.2**	**y = −0.15x + 18**	**y = −0.01x + 1.57**	**y = −1.0x + 113.3**	**y = 0.003x + 0.5**	**y = −0.03x + 7.6**	**y = 0.162x + 42.7**	**y = −0.37x + 80.5**
R^2^	**0.064**	**0.03**	**0.06**	**0.07**	**0.48**	**0.40**	**0.23**	**0.240**	**0.02**	**0.09**	**0.04**
p-level	**0.000**	**0.001**	**0.000**	**0.000**	**0.000**	**0.000**	**0.000**	**0.000**	**0.006**	**0.000**	**0.000**

a, b, c, d, e indicates the differences between depths, statistically significant at p < 0.05.

Average of the soil properties showed that the bulk density in the deep soils was 1.06-fold higher than the surface soils. The soil water was peaked at the 20–40 cm (14.34%). The pH values in the bottom soils were 1.06-fold higher than that in the surface soils. The peak EC at the surface soils was 1.7-fold higher than that in the deep soils. The peak SOC at the surface soils was 3.4-fold higher than that in the 80–100 cm soil, while nearly 3-fold differences between the surface and deep soils were observed in the N, AN and P. About 1.5-fold differences between the surface and deep soils were observed in the AK and AP (Table 2).

Stepwise regressions found that the glomalin amounts in the surface soils and the deep soils were regulated differently by the soil properties, soil nutrients and climate conditions in sampling sites (Table 3). Both the surface soils and the deep soils showed that the higher SOC, together with the lower values in pH and MAT was in line with the higher EEG amounts in the surface soils, and R^2^ in the deep soils was 1.3-fold higher than that in the surface soils (Table 3). In the case of TG, both the surface soils and the deep soils showed that the higher SOC accompanied with the higher TG; However, the stepwise model in the surface soils included climatic conditions (MAT and MAP), while these two parameters were not found in the deep soils and as instead, pH entered in the deep soil’s model (Table 3).

**Table 3:** Stepwise regressions between glomalin features and various soil properties, and their differences at different soil layers (Stepwise regression: F-enter probability value was at p < 0.01, and F-removal probability value was at p > 0.05). The constants in the models were not listed in the table for minimizing the size of the table.

Y	Parameters	Surface soils 0–40 cm				Deep soils 40–100 cm				
		Unstandard B	Standard Beta	Sig	R^2^	Parameters	Unstandard B	Standard Beta	Sig.	R^2^
EEG	SOC	0.02	0.41	0.00	0.53	pH	−0.26	−0.59	0.00	0.69
	MAT	−0.18	−0.31	0.00		MAT	−0.17	−0.28	0.00	
	pH	−0.27	−0.60	0.00		EC	−0.001	−0.17	0.00	
	Altitude	−0.001	−0.29	0.00		Altitude	−0.001	−0.26	0.00	
	water	−0.01	−0.17	0.01		SOC	0.026	0.32	0.00	
TG	SOC	0.20	0.50	0.00	0.63	SOC	0.32	0.60	0.00	0.62
	MAP	0.06	0.60	0.00		pH	−1.28	−0.43	0.00	
	water	−0.17	−0.36	0.00		water	−0.07	−0.17	0.00	
	MAT	−1.46	−0.34	0.00						
EEG/SOC	SOC	−0.001	−0.60	0.00	0.48	MAT	−0.009	−0.28	0.00	0.53
	MAT	−0.007	−0.41	0.00		water	−0.001	−0.25	0.00	
	pH	−0.005	−0.35	0.00		pH	−0.015	−0.59	0.00	
						MAP	0.000	−0.33	0.00	
						SOC	−0.001	−0.18	0.00	
TG/SOC	SOC	−0.004	−0.45	0.00	0.24	pH	−0.070	−0.48	0.00	0.30
	MAP	0.001	0.616	0.00		water	−0.007	−0.34	0.00	
	water	−0.004	−0.34	0.00		SOC	−0.005	−0.19	0.01	
	MAT	−0.029	−0.29	0.00						
EEG/N	N	−0.01	−0.50	0.00	0.58	MAT	−0.018	−0.48	0.00	0.55
	MAT	−0.009	−0.38	0.00		EC	−7.8E-5	−0.17	0.00	
	pH	−0.006	−0.34	0.00		N	−0.013	−0.27	0.00	
	water	−0.001	−0.28	0.00		pH	−0.011	−0.36	0.00	
						water	−0.001	−0.23	0.00	
TG/N	N	−0.120	−0.80	0.00	0.40	pH	−0.071	−0.43	0.00	0.38
	SOC	0.007	0.49	0.00		N	−0.135	−0.48	0.00	
	pH	−0.022	−0.18	0.01		water	−0.006	−0.26	0.00	
						SOC	0.010	0.34	0.00	
						K	0.001	0.18	0.00	
EEG/P	P	−0.050	−0.45	0.00	0.18	P	−0.146	−0.24	0.00	0.06
	SOC	0.001	0.28	0.00						
TG/P	P	−0.314	−0.47	0.00	0.21	P	−0.972	−0.26	0.00	0.07
	SOC	0.007	0.33	0.00						
EEG/TG	SOC	−0.006	−0.44	0.00	0.31	MAT	−0.19	−0.37	0.00	0.17
	MAT	−0.057	−0.38	0.00		SOC	−0.01	−0.17	0.01	
Statistics	climatic entered/total	9/29 = 31.0%		6/30 = 20.0%						
	physiochemical entered/total	8/29 = 27.6%		13/30 = 43.3%						
	nutrient entered/total	12/29 = 41.4%		11/30 = 36.7%						
	SOC times/ |average|	8/0.44			6/0.30					
	pH times/ |average|	4/0.37			6/0.48					
	EC times/ |average|	0/0			2/0.17					
	MAT times/ |average|	6/0.30			4/0.35					

Note: water is soil water percentage. SOC times mean the times of SOC entering the stepwise models, pH, EC and MAT times had the similar meaning. EEG/SOC, relative ratio of the carbon in EEG to total SOC; TG/SOC, relative ratio of the carbon in TG to total SOC; EEG/N, relative ratio of the nitrogen in EEG to total soil N; TG/N, relative ratio of the nitrogen in TG to total soil N; EEG/P, relative ratio of the phosphorus in EEG to total soil P; TG/P, relative ratio of the phosphorus in TG to total soil P; EEG/TG, relative ratio of the EEG to TG.

In the case of glomalin contribution to total SOC (glomalin/SOC), the lower SOC, pH, and MAT accompanied with the higher EEG/SOC and TG/SOC; However, the standard coefficient (beta) for the stepwise regressions showed that the SOC’s influences in the surface soils (−0.45 to −0.60) were about 3-fold higher than those in the deep soils (−0.18 to −0.19). Moreover, the pH’s influences in the deep soils (−0.48 to −0.59) were usually much higher than those in the surface soils (0 to −0.35). Total climatic influences in the surface soils (MAT’s beta, −0.29 to −0.41) were similar to those in the deep soils (−0.28 in the deep soils) (Table 3).

In the case of glomalin contribution to total N (glomalin/N), the stepwise model included parameters of N, pH, MAT, SOC and soil water. In general, the lower N, pH, and MAT usually accompanied with the higher EEG/N in the surface soils and the deep soils, however, the N’s standard coefficients in the surface soils (beta = −0.50) were about 2-fold higher than that in the deep soils (beta = −0.27). For a higher TG/N both in the surface and deep soils, there were accompanying lower values in N, pH but higher SOC; Beta values for N and SOC in the surface soils were much larger than did in the deep soils, while the beta value for pH showed a contrary tendency (Table 3).

In the case of glomalin contribution to total P (glomalin/P), the surface soils showed that the lower P but higher SOC accompanied with the larger EEG/P and TG/P; However, in the deep soils, only soil P entered the stepwise model. Standard coefficient values (beta values) showed that the P’s effects in the surface soils were about 2-fold higher than those in the deep soils (Table 3). Both the surface soils and the deep soils showed that the higher SOC and MAT usually accompanied with the lower EEG/TG, and the SOC’s beta values for the surface soils were about 2.5-fold higher than did in the deep soils, while the MAT’s beta value in the surface soils (−0.38) was the same to that in the deep soils (−0.37) (Table 3).

In all, the statistics of whole stepwise regressions showed that in the surface soils, glomalin features were more regulated by soil nutrients, as manifested by the 41.4% parameters as soil nutrients into the stepwise models compared with the much lower percentage in the deep soils (36.7% parameters were soil nutrients); However, soil physiochemical properties greatly regulated glomalin features in the deep soils (43.3% parameters as soil physiochemical properties) compared with those at the surface soils (27.6% parameters as soil physiochemical properties) (Table 3).

Of the parameters, the SOC and pH were the most frequent parameters related to soil nutrients and soil physiochemical properties, while MAT was the most frequent climatic parameter. In the surface soils, SOC entering 8 times and pH entering 4 times were observed in the stepwise models, while in the deep soils, more pH (6 times) but less SOC (6 times) entered the models. The standard coefficient beta averages for SOC and pH at the surface soils were 0.44 and 0.37, and in the deep soils they were 0.30 and 0.48, showing an opposite tendency between the surface and deep soils in their regulation of glomalin-related features (Table 3).

### Pearson correlation difference between the surface and the deep soils

Pearson correlations were also used to check the different associations between glomalin and various factors in the deep soils comparison with the surface soils (Table 4). In the case of soil physiochemical property-glomalin correlations, the coefficient ratio for the deep soils/surface soils for bulk density, soil pH and EC were generally higher than 1.0, showing larger coefficients were usually found in the deep soils (Table 4).

**Table 4:** Pearson correlations between glomalin features and soil physiochemical properties, nutrients and climatic conditions, and differences between the surface and deep soils.

Parameter	depth	EEG	TG	EEG/SOC	TG/SOC	EEG/N	TG/N	EEG/P	TG/P	EEG/TG
Soil physicochemical properties, stronger regulations at the deep soils than the surface soils										
Bulkdensity	Surface	−0.100	−0.248^**^	0.313^**^	0.105	0.356^**^	0.160	0.065	0.025	0.240^**^
	Deep	−0.012	−0.255^**^	0.364^**^	0.053	0.395^**^	0.092	0.123	0.070	0.269^**^
	D/S	—	1.03	1.17	—	1.11	—	—	—	1.12
soilwater	Surface	−0.010	0.075	−0.224^**^	−0.168^*^	−0.446^**^	−0.332^**^	−0.010	0.025	−0.124
	Deep	0.213^**^	0.192^**^	−0.267^**^	−0.330^**^	−0.168^*^	−0.200^**^	0.056	0.029	0.088
	D/S	21.3	2.56	1.19	1.96	0.37	0.60	—	—	—
pH	Surface	−0.396^**^	−0.338^**^	−0.147	−0.204^*^	−0.169^*^	−0.179^*^	0.060	0.064	0.110
	Deep	−0.644^**^	−0.592^**^	−0.315^**^	−0.341^**^	−0.344^**^	−0.413^**^	0.017	0.026	0.044
	D/S	1.63	1.75	2.14	1.67	2.04	2.31	—	—	—
EC	Surface	−0.094	−0.011	−0.176^*^	−0.130	−0.068	−0.043	−0.043	−0.035	−0.115
	Deep	−0.419^**^	−0.315^**^	−0.391^**^	−0.329^**^	−0.414^**^	−0.357^**^	−0.085	−0.085	−0.063
	D/S	4.46	28.6	2.22	2.53	6.09	8.30	—	—	—
Soil nutrients, generally lower regulations in the deep soils than the surface soils.										
SOC	Surface	0.427^**^	0.682^**^	−0.517^**^	−0.262^**^	−0.230^**^	0.002	0.099	0.147	−0.421^**^
	Deep	0.414^**^	0.659^**^	−0.280^**^	−0.185^**^	−0.174^*^	−0.046	−0.069	−0.028	−0.177^**^
	D/S	0.97	0.97	0.54	0.71	0.76	—	—	—	0.42
N	Surface	0.261^**^	0.432^**^	−0.370^**^	−0.209^*^	−0.583^**^	−0.456^**^	−0.011	0.031	−0.267^**^
	Deep	0.323^**^	0.577^**^	−0.257^**^	−0.101	−0.333^**^	−0.228^**^	−0.070	−0.024	−0.192^**^
	D/S	1.23	1.34	0.69	0.48	0.57	0.50	—	—	0.72
AN	Surface	0.247^**^	0.314^**^	−0.263^**^	−0.181^*^	−0.220^**^	−0.143	−0.021	0.001	−0.114
	Deep	0.107	0.291^**^	−0.104	0.056	−0.097	0.046	0.000	0.015	−0.088
	D/S	0.43	0.93	0.38	0.31	0.44	—	—	—	—
AP	Surface	0.366^**^	0.235^**^	0.175^*^	0.139	0.268^**^	0.200^*^	−0.001	−0.021	0.031
	Deep	0.333^**^	0.327^**^	0.053	0.147^*^	0.072	0.168^*^	−0.070	−0.068	−0.095
	D/S	0.91	1.39	0.30	1.06	0.27	0.84	—	—	—
P	Surface	0.153	0.330^**^	−0.186^*^	−0.050	−0.232^**^	−0.079	−0.34^**^	−0.34^**^	−0.208^*^
	Deep	0.128	0.294^**^	−0.133	−0.025	−0.131	−0.008	−0.24^**^	−0.26^**^	−0.159^*^
	D/S	—	0.89	0.72	—	0.56	—	0.71	0.76	0.76
K	Surface	0.042	−0.057	0.130	−0.002	0.129	0.035	0.086	0.073	0.105
	Deep	0.118	0.058	0.268^**^	0.260^**^	0.306^**^	0.303^**^	0.004	−0.028	0.009
	D/S	—	—	2.06	130	2.37	8.66	—	—	—
AK	Surface	0.178^*^	0.240^**^	−0.090	−0.039	−0.114	−0.064	−0.036	−0.027	−0.105
	Deep	0.104	0.106	−0.131	−0.156^*^	−0.107	−0.128	0.036	0.030	0.008
	D/S	0.58	0.44	—	4.00	—	—	—	—	—
Climatic conditions, at least similar influences in the surface and deep soils										
MAT	Surface	−0.349^**^	−0.039	−0.297^**^	0.068	−0.276^**^	−0.026	−0.141	−0.080	−0.353^**^
	Deep	−0.444^**^	0.054	−0.414^**^	0.114	−0.477^**^	−0.008	−0.131	−0.046	−0.371^**^
	D/S	1.27	—	1.39	—	1.73	—	—	—	1.05
MAP	Surface	0.123	0.500^**^	−0.314^**^	0.133	−0.348^**^	0.023	−0.148	−0.065	−0.451^**^
	Deep	0.136^*^	0.526^**^	−0.345^**^	0.109	−0.274^**^	0.176^*^	−0.116	−0.059	−0.313^**^
	D/S	1.11	1.05	1.10	—	0.79	7.65	—	—	0.69
Altitude	Surface	−0.031	0.256^**^	−0.201^*^	0.129	−0.185^*^	0.046	−0.125	−0.082	−0.337^**^
	Deep	0.051	0.429^**^	−0.198^**^	0.157^*^	−0.165^*^	0.185^**^	−0.081	−0.045	−0.236^**^
	D/S	—	1.67	0.99	1.22	0.89	4.02	—	—	0.70

Note: The same to Table 1. ** means that the coefficient was significant at *p* < 0.01, while * means that the coefficient was significant at *p* < 0.05. D/S means the ratio between deep soil to surface soil in the correlation coefficient.

In the case of nutrient-glomalin correlations, different patterns were found, i.e, general lower coefficients at the deep soils were observed compared with those in the surface soils. For example, SOC-glomalin coefficients, AN-glomalin coefficients, and P-glomalin coefficients in the deep soils were lower than those in the surface soils. While most of N, AP and AK showed the similar tendency, except K showed a contrary tendency (Table 4).

Climatic-glomalin correlations showed general similar coefficients between the surface and deep soils, while different climatic parameters showed different patterns. For example, MAT-glomalin coefficients in the deep soils were 1.05–1.73 fold higher than those in the surface soils. Four MAP-glomalin coefficients showed the higher values in the deep soils, while the other 3 coefficients showed the higher values in the surface soils (Table 4).

### RDA variation partitioning and difference between the surface and deep soils

**Figure 1:**
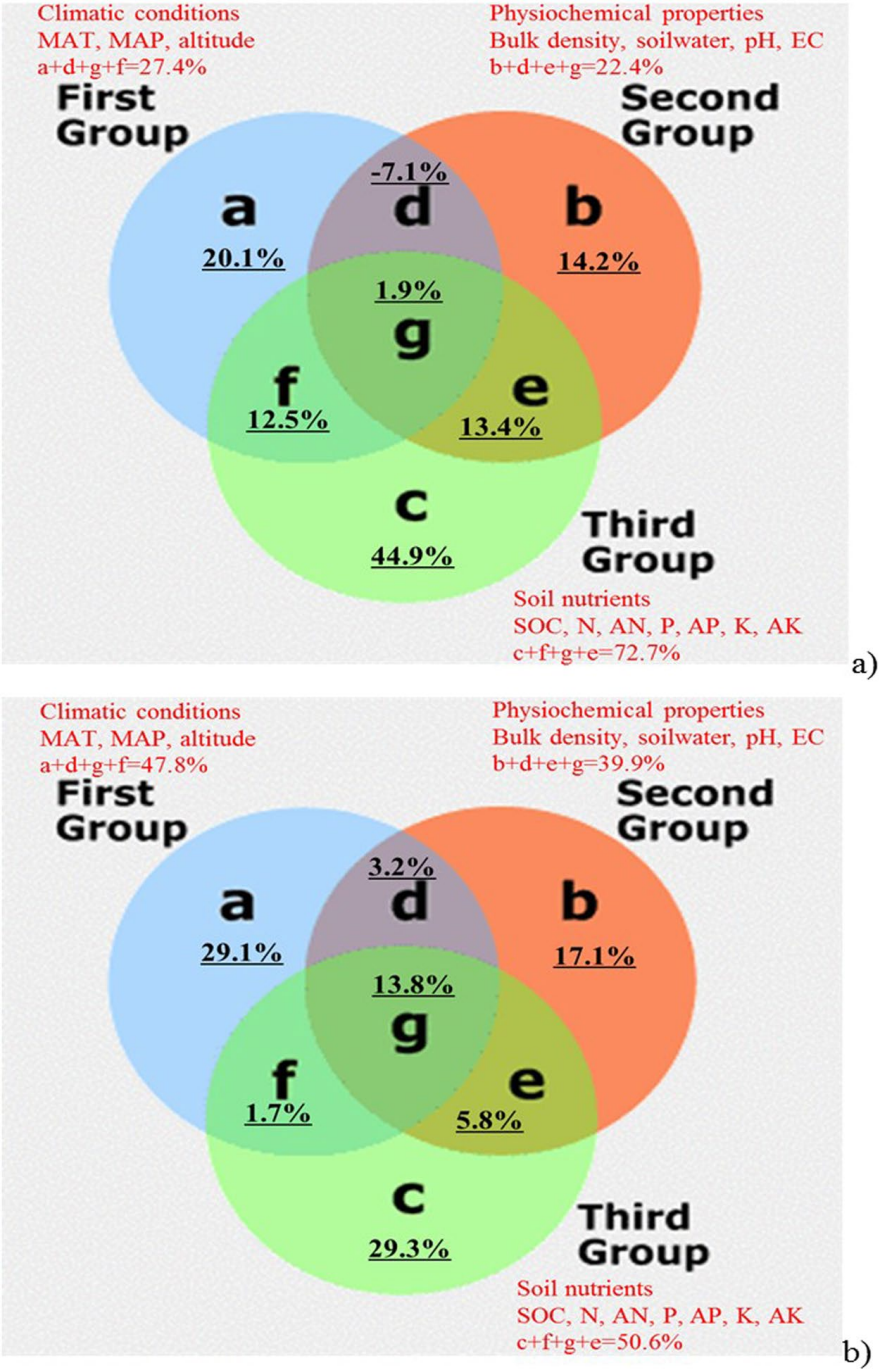
RDA ordination-based glomalin variation partitioning at the surface soils (**a**) and the deep soils (**b**). In the case of the surface soil, soil nutrients are responsible for more glomalin variation (44.9% for the unique effect; 72.7% for the pooled effect) than in the deep soils (29.3% for the unique effect; 50.6% for the pooled effect); However, physiochemical properties of soil are responsible more variation in the deep soils (17.1% for the unique effect; 39.9% for the pooled effect) compared with the surface soils (14.2% for the unique effect; 22.4% for the pooled effect). Climatic conditions showed important effects in regulating glomalin accumulation in both the surface (20.1% for the unique effect) and the deep (29.1% for the unique effect) soils.

By using the total variations of glomalin parameters as a whole (100%), RDA variation partitioning results showed that in the surface soils, the unique effect from soil nutrients contributed 44.9% (c) of the glomalin variations, while the deep soils showed a lower percentage (c, 29.3%). In contrary, glomalin variations in the deep soils could be explained more by unique effects from climatic conditions (a, 29.1%) and soil physiochemical properties (b, 17.1%), than did in the surface soils (a, 20.1% and b, 14.2% respectively) (Fig. 1). In the surface soils, interactions of climatic conditions, soil physiochemical properties and soil nutrients (d+e+f+g) were responsible for 20.7% of the variations, and in the deep soils, they contributed 24.5% of the variations (Fig. 1).

Pooling the data together, we found the similar tendency in total effects. For example, at the surface soils, total explaining percentages from climatic conditions (a+d+g+f) and soil physiochemical properties (b+d+g+e) were 27.4% and 22.4%, respectively; However, the explaining percentages in the deep soils became larger (47.8% and 39.9%, respectively). In contrary, explaining percentage from soil nutrients (c+e+f+g) in the surface soils (72.7%) was much larger than did in the deep soils (50.6%) (Fig. 1).

### SEM analysis on the causal relations for glomalin changes: direct and indirect effects

**Figure 2:**
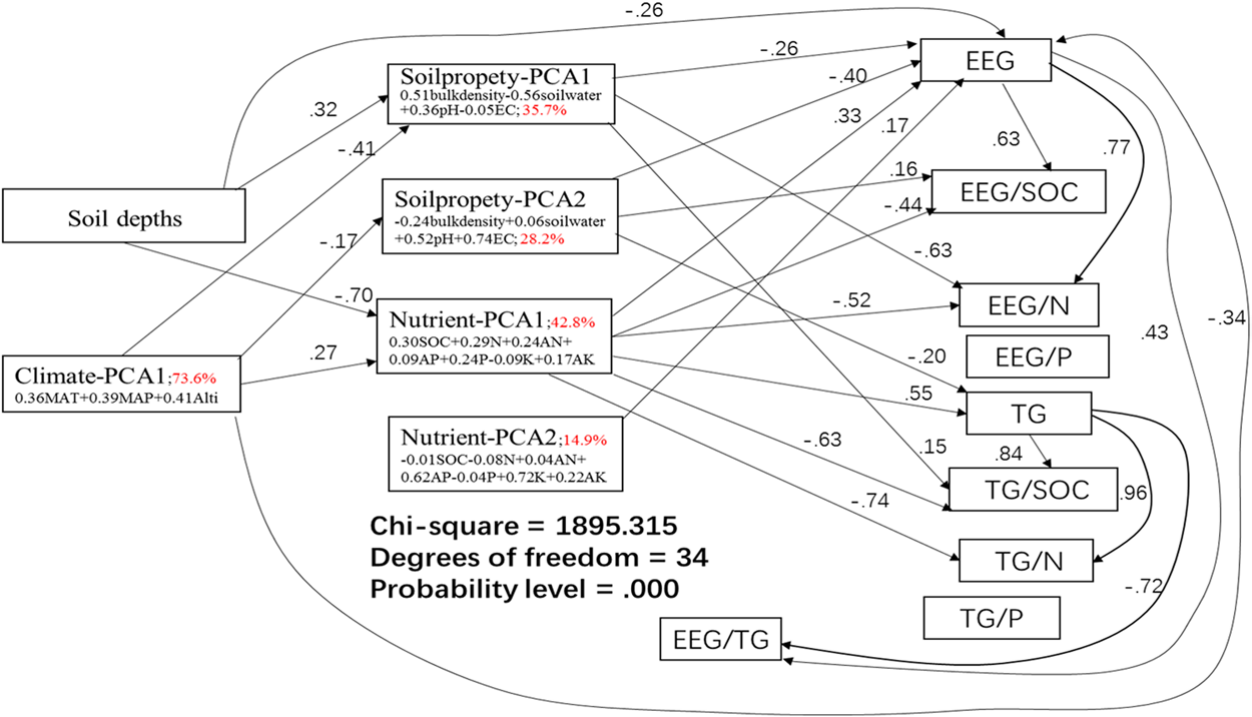
SEM analysis on the causal relations between soil depths, climate, soil nutrients and physiochemical properties and glomalin features. The significant direct coefficients were shown in the figures, while non-significant ones were not shown here. The regression weights with a statistical significance *p* < 0.0001 were listed in the figures. PCA1 and PCA2 are the first and second principal component for climatic conditions(climatic-PCA), soil physiochemical properties (soilproperty-PCA) and soil nutrients(nutrient-PCA). EEG/SOC, relative ratio of the carbon in EEG to total SOC; TG/SOC, relative ratio of the carbon in TG to total SOC; EEG/N, relative ratio of the nitrogen in EEG to total soil N; TG/N, relative ratio of the nitrogen in TG to total soil N; EEG/P, relative ratio of the phosphorus in EEG to total soil P; TG/P, relative ratio of the phosphorus in TG to total soil P; EEG/TG, relative ratio of the EEG to TG.

A PCA method was used to extract the main information from the data. In the climatic conditions, one main component was derived, and 73.6% of the variation was included in the main component (climatic-PCA1) (Table A1; Fig. 2). In climatic-PCA1, MAT, MAP and altitude showed the similar contribution as shown in the quite similar standard coefficient (0.36–0.41) (Fig. 2; Table A2). Soil physicochemical properties component analysis showed that 2 main component extracted 63.9% information from the raw data (soilproperty-PCA1, 35.7%; soilproperty-PCA2, 28.2%). Soilproperty-PCA1 included information from bulk density (0.51) and soil water (−0.56); while soilproperty-PCA2 mainly included information from EC (0.74) and pH (0.52) (Fig. 2; Table A2). Two main components from soil nutrients extracted total 57.6% information from the raw data. Of them, nutrient-PCA1 contributed 42.8% and nutrient-PCA2 contributed 14.9% of the variations (Table A1; Fig. 2). Nutrient-PCA1 extracted information mainly from SOC (0.30), N (0.29), AN (0.24) and TP (0.24); while nutrient-PCA2 extracted information mainly from K (0.72), AP (0.62) and AK (0.22) (Fig. 2; Table A2).

As shown in Fig. 2 and Table A3, a direct effect of soil depth influence on the glomalin features were only found in EEG (−0.26), while all other vertical differences were from the indirect effects via soil physiochemical properties and soil nutrients. For example, the deeper soils directly resulted in the higher soil bulk density but the lower soil water (soil propertyPCA1, 0.32), which finally resulted in the lower EEG (−0.26), EEG/N (−0.63) but the higher TG/SOC (0.15), and the indirect coefficients for these influences were respectively −0.08 (=0.32*−0.26), −0.20 (=0.32*−0.63) and 0.05 (=0.32*0.15). The indirect effects might also be achieved from the soil nutrientPCA1 (mainly SOC and N) (Fig. 2), i.e., the deeper soils could directly result in a lower SOC and N (−0.70), and this nutrient shortage in the deep soils resulted in the lower glomalin (0.33 for EEG and 0.55 for TG), but the higher glomalin contribution to SOC (−0.44 for EEG/SOC, −0.63 for TG/SOC) and N (−0.52 for EEG/N, −0.74 for TG/N); The corresponding indirect effects were −0.23 to −0.39 for glomalin (EEG and TG), 0.31 to 0.44 for glomalin/SOC, 0.36 to 0.52 for glomalin/N (Fig. 2).

Climatic conditions at the sampling sites also directly regulated the EEG accumulation (−0.34), but no effects on the TG, showing that the higher MAT, MAP and altitude were in line with the lower EEG accumulation in soils. Climate could indirectly affect the glomalin features through its significant influences on the soil propertyPCA1 (bulk density and soil water), the soil propertyPCA2 (EC and pH) and the soil nutrientPCA1 (SOC and N), but not through the nutrientPCA2 (K and AP) (Fig. 2). The higher climate-PCA1 value directly resulted in the lower soilpropertyPCA1 (i.e., a higher soil water but a lower soil bulk density, coefficient −0.41), and the lower soilpropertyPCA2 (i.e., a lower EC and pH, coefficient −0.17), but the more fertile soils (i.e., a higher SOC and N, coefficient 0.27); These changes in soil physiochemical properties and nutrients resulted in glomalin changes directly (Fig. 2; Table A3).

In all, SEM results manifested that the soil depth and the climatic condition could directly affect EEG accumulation, while its effects on glomalin contribution to SOC, N and TG indirectly occurred through their strong associations with soil physiochemical properties and soil nutrients related with SOC and N (Fig. 2; Table A3).

## Discussion

### Lower glomalin amounts but larger contribution to carbon sequestration and nutrient storage in the deep soils

In this paper, we found that much lower glomalin accumulated in the deep soils, while their contribution to SOC and nutrients in the deep soils was 1.1–1.3-fold higher than did in the surface soils, showing the importance glomalin-related carbon and nutrients in the deep soils compare with those in the surface soils. Previous studies also found 3.1–3.3-fold higher glomalin in the surface soils, compared with the deep soils in farming-pastoral zones^36^. In a shallow 40 cm soil profile, soil glomalin exhibits obvious vertical distribution pattern, which decreases with increasing soil depth, and soil glomalin is significantly directly related with soil available phosphorus and protease^19^. The contribution of glomalin to dissolve organic carbon differed in land uses and seasonality in dry tropics, and the ratio of glomalin-C to dissolve organic carbon was lower in forests than fallow lands and agriculture lands^37^. Deep soil organic matter is a key but poorly understood component of terrestrial C cycle, and generally, C in the deep soil horizons is characterized by high mean residence times of up to several thousand years^38^. GRSP composition was a rich mixture of proteinaceous, humic, lipid and inorganic substances^39^, and is extremely stable in nature^1,2^. SOM in subsoils is enriched in microbial-derived C compounds and could be depleted in energy-rich plant material compared to topsoil SOM^38^. Consequently, the increased fraction of glomalin-C to SOC with increasing sampling depth might provide new evidence that microbial derived C, especially AMF-C, promoted SOC accumulation in the subsoil^9^. One of the most important factors leading to protection of SOM in subsoils may be the spatial separation of SOM, microorganisms and extracellular enzyme activity possibly related to the heterogeneity of C input^38^. Our findings indicate that the higher contribution of glomalin to SOC in the deep soils may possibly contribute the stronger stability of SOC in the deep soils.

GRSP is a glycoprotein with a major asparagine-linked (N-linked) chain of carbohydrates, tightly bound with iron together with organic matter and amino acids^13^, possibly an important proxy of AMF amounts in soils^40^. Deep soil carbon and nutrient depletion have been observed in forests in NE China^34^, and also in other different studies, such as Amazonian forests^41^, reforestation on grasslands^42^ and croplands^43^. Most of the glomalin compositional traits were stable with soil physiochemical changes^14,44^ showing that this kind of glomalin-related nutrients may possibly restrict the nutrient supply for subsoil microbial activities. Actually, the absence of fresh organic carbon, an essential source of energy for soil microbes, the stability of organic carbon in deep soil layers is maintained^20^. The much higher nutrient allocation in glomalin is a future of the deep soil nutrients, and this is a supplement for previous studies.

### Lower nutrient- but higher physiochemical-regulations on glomalin in the deep soils

How to identify the possible factors affecting GRSP changes still have challenges in a field sampling campaign, and statistical methods including regression analysis, redundancy ordination, PCA and SEM analysis were used in this paper, which has been proved beneficial for determining the causal relationship between patterns^26^. Accordingly, we confirmed that different glomalin-regulating mechanism between the surface soils and the deep soils. In the surface soils, glomalin was regulated more by soil nutrients (mainly SOC and N), while soil physiochemical properties (e.g. pH) more greatly regulated glomalin features in the deep soils. Variation partitioning from RDA ordinations showed 17.5% more glomalin variations could be explained by soil physiochemical properties at the deep soils, while 22.1% more variations could be explained by soil nutrients in the surface soils (Fig. 1). The importance of soil depth in regulating glomalin regulations could be manifested by two aspects via the SEM analysis. The first is its direct influences on EEG accumulation; The second is the strongly indirect effects through the depth-dependent soil nutrients and physiochemical changes (Fig. 2). The general tendency is that the higher soil nutrients, the lower values in soil bulk density, soil pH and EC accompanied with the higher glomalin accumulation in soils, but lower glomalin contribution to the SOC and N storage. With soil depth deepened, less EEG could be accumulated, but much slight influences on TG.

Deep soil differences from the surface soils have been often reported in previous studies. Occlusion within soil aggregates has been identified to account for a great proportion of SOM preserved in subsoils^38^. Deep soil horizons are recognized great contribution and importance to soil carbon pools, thus are also important in assessing whole-ecosystem response to management and global change^18^, possibly through the close relation between SOC vertical distribution and local climate^45^ or deep soil water in modulating climate^46^. The surface-deep soil differences in factors regulating glomalin distribution, i.e., lower nutrient (SOC)- but higher physicochemical (pH)-regulations on glomalin in the deep soils were found when compared with the surface soils, provided a new evidence of the deep soil organic matter importance for terrestrial C and nutrient cycle from the view point of AMF and glomalin contributions^34,38^. The soil conditioning function of GRSP has well reported, and understanding of how to improve GRSP accumulation in soil is important for the GRSP-oriented degraded soil rehabilitation^14,44^. Long-term land uses could affect fractions of glomalin and SOC in the Indo-Gangetic plain^10^. The primary forests had 2.35–2.56-fold higher GRSP amount than those in the plantation forests and farmlands^14^, while the GRSP amount significantly correlated with soil bulk density and soil water^47^. However, from land uses changes were not well-defined to date. Our paper gave a supplement on this data shortage on the underlying forming mechanism for glomalin changes.

### Similar climatic regulations on glomalin in the surface and deep soils

The northeastern China is a sensitive region of climate change, and potential evapotranspiration (PE) and moisture index (MI) based on the observation data of 1961–2004 from 94 meteorological stations showed a general increasing tendency in MAT, MAP, PE and MI^48^. Moreover, an increasing tendency was more significant in MAT and PE than in MAP and MI^48^, showing that northeast China is the most serious region processing in global changes, especially temperature warming. Although the carbon sink capacity of the forest ecosystems in Northeast China has been weakened since 2003, the total carbon absorption will keep increasing, and will play a positive role in the mitigation of climate change from the significant carbon sink capacity^49^. Underground soil changes during the global warming process were still in controversial, particularly the AMF and glomalin-related processes, although a previous study has found that GRSP could response to elevated CO_2_ and N addition in a subtropical forest, which favors the potential consequences for soil C accumulation^9^.

In this paper, we found that climatic warming could possibly decrease the glomalin accumulation in soils, EEG/TG ratio and glomalin contribution to SOC and nutrients (Table 3 and Table 4). Moreover, the SEM analysis showed that EEG was directly down-regulated by climatic changes (warming, precipitation and altitude), while the others were indirectly affected from the climatic influencing on soil properties and soil nutrients (Fig. 2). Both stepwise regression statistics, Pearson correlation and RDA-variation partitioning clearly manifested that the climatic change influences on glomalin in the deep soils were similar to those in the surface soils. Moreover, the interactions between climatic changes, soil physiochemical properties and soil nutrients also largely regulating the glomalin accumulation both in the surface and deep soils. Until recently, the properties and dynamics of C and nutrients in the deep soils were largely ignored^38^. Forest growth could result in SOC accumulation, taking a large percentage of whole forest ecosystem carbon sequestration^34,50^; Moreover, deep soils showed a contrary tendency than did in the surface soils in nutrient dynamics^34,50^. Our findings indicate that the climatic influences on glomalin accumulation can reach as deep as 100 cm in soil depth, and possible underlying reason should be related with soil-mycorrhizal interactions. Mycorrhiza’s effects on soils were possible from particle size, surface structure, mineral crystallinity, functional groups, and elemental composition of soil colloids and enzyme interactions^51–53^. Microbial priming effects are possibly related to the vertical patterns since the supply of new carbon into subsoils could stimulate the microbial mineralization of 2,567-year-old carbon^20^. In future studies, glomalin changes in the deep soils together with AMF diversity should be focused in studying soil processes in climatic changes.

### Glomalin importance both in the surface and deep soils: implications from this study

The United Nations has completed the first-ever global assessment of the state of the planet’s land resources in late 2011, finding that a quarter of all farmland is highly degraded and warning the trend must be reversed if the world’s growing population is to be fed (http://www.theage.com.au/). In China, the grain production in Songnen Plain accounts for over 10% of total commercial grain output in China^54^, owing to fertile black soils in this region^55,56^. However, soil degradation restricts agricultural development^57,58^ as shown be a 50% reduction in SOM and soil fertility^59^, serious saline-alkalinization^60^ and soil physical degradation of soil bulk density increase and decrease of total porosity^50^. The following suggestions for northeastern China farmland development could be proposed based on the results of this study.

Firstly, the underlying mechanism in regulating glomalin accumulation in the surface soils provide a basis for degraded soil improvement, and soil organic amendments and acidity adjustments should be possible measures owing to its strongly regulating on glomalin accumulation. Previous studies have manifested that the SOC decreases and bulk density increases closely associated with the declines in GRSP amounts and compositional traits^14,40,44,58^, and some active steps should be supplemented for improving GRSP accumulation for securing their soil conditioning function^14,61^. As shown in this study, SOC is the top important regulator for glomalin accumulation in the surface soils, thus, future measures to improve glomalin in farming soils should consider more organic material returning to the soils. For example, organic manure amendments should be highlighted, and some studies also have shown that redistribution of glomalin in macroaggregates possibly contributed to soil aggregates stability^62^. Long-term organic fertilization practices could alter soil ergosterol content, glomalin, and phospholipid fatty acid profile^63^. By using glomalin as the activity of AMF, over 20-yr survey showed that glomalin increased under no-till and organic management owing to changes in AMF communities^64^. Organic, unlike conventional practices, promoted AM root colonization in apple, and possibly associated with soil pH, P, Zn, Mn, C and leaf P, Ca, Mg and tree growth^65^. Furthermore, soil pH was also very important for the glomalin accumulation. At present, a total of 3.73 million hm^2^ saline-sodic lands distributes in the Songnen Plain and saline-alkalization becomes more serious under global warming process in this region^66^. Anti-measures to decline the processes of saline-alkalization will benefit the accumulation of glomalin in soils, such as anti-agent additions^60^ or suitable species selections for acidifying soils^67^.

Secondly, the much stronger regulations on the deep soil glomalin suggested that future evaluation of glomalin and AMF importance should fully consider deep soils. New mechanical understanding of AMF and soil properties relations have been proposed, such as Clemmensen, *et al*.^68^’s mathematical partitions of fungi-C from soil C, as well as Cardoso and Kuyper^69^’s fungus-assistance for plant P transporters with indirect effects on N availability. Deep soil importance has been highlighted by the review paper^38^ and some research paper^34^. In this paper, we confirmed that regulating mechanism of glomalin accumulation differed from the surface and deep soils, and they should be related with the climatic, soil physiochemical and nutrient adjustment. In the future assessment of glomalin importance should include both the surface and deep soils together for an exact evaluation.

## Conclusion

GRSP showed obvious vertical changes in both its amounts, glomalin/SOC, glomalin/nutrients, EEG/TG, and a lower glomalin amounts but a larger contribution to carbon sequestration and nutrient storage in the deep soils were generally observed. Soil physicochemical properties, soil nutrients and local climates closely associated with these vertical glomalin variations. The different statistical analysis confirmed that lower nutrient- but higher physiochemical-regulations on glomalin features were in the deep soils compared with those in the surface soils (mainly SOC and pH-related regulation), while the similar climatic regulations on glomalin were found on the surface and deep soils. Our results highlight the importance of soil fertility, SOC and pH in regulating glomalin characteristics, and these regulations were depth-dependent. These findings provide a basis for GRSP-oriented degraded soil recovery in black soil region in northeastern China, as well as scientific evaluations of glomalin and AMF importance in whole soil profile into 1 m depth.

## Materials and Methods

### Study areas and soil sampling

**Figure 3:**
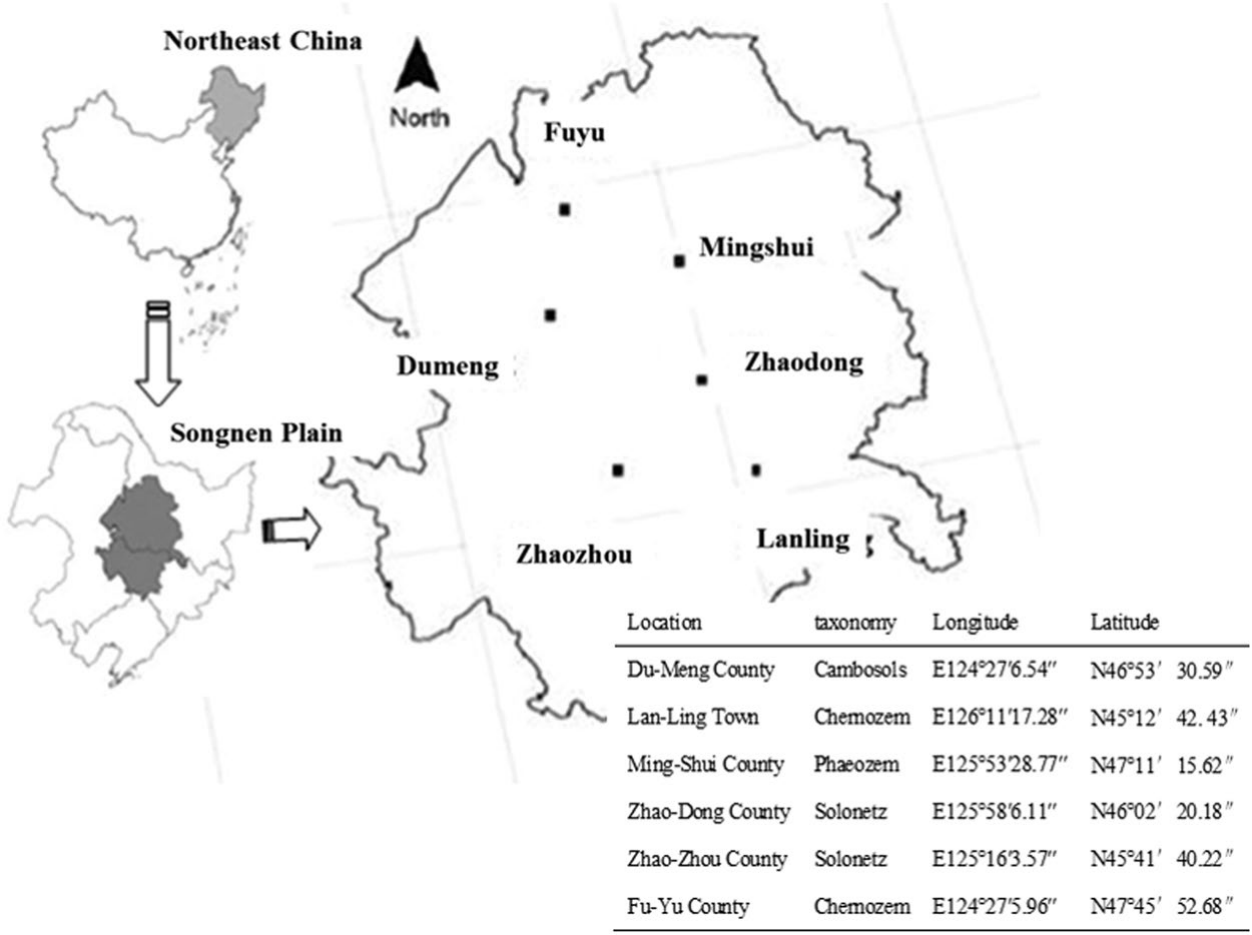
Distribution of sampling locations across the Songnen Plain in northeastern China and their basic information. The map was generated by software ArcGIS 10.0 (www.arcgis.com/) by Zhong Zhaoliang.

Soil samples were collected from 6 different locations randomly selected in farmlands at Songnen Plain, northeastern China. The 6 locations are Du-Meng (mean annual temperature [MAT] 3.1 °C; mean annual precipitation [MAP] 421 mm; altitude 147 m), Lan-Ling (MAT 4.4 °C; MAP 481 mm; altitude 381 m), Ming-Shui (MAT 2.9 °C; MAP 480 mm; altitude 259 m), Zhao-Dong (MAT 3.8 °C; MAP 467 mm; altitude 166 m), Zhao-Zhou (MAT 3.5 °C; MAP 450 mm; altitude 147 m), and Fu-Yu (MAT 3.0 °C; MAP 441 mm; altitude 158 m) (Fig. 3). At the sampling time, the crop at the sampling sites was maize, and previous crops were maize and soybean for many years. According to the Chinese Soil Classification System, the soil types in the study region are typical black soils, including Chernozem, Phaeozem, and Cambisols, and some degraded soil, such as Solonetz. According to the USDA Classification System, these belong to Mollisols, Entisols and Aridisols. In Dumeng, soil texture was sand (73%), silt (17%) and clay (10%); In Fu-Yu, sand was 42%, silt and clay were 34% and 24%, respectively; In Lan-Ling and Ming-Shui, sand were 19%, silt and clay were 55–58% and 22–25%, respectively. In Zhao-Dong and Zhao-Zhou, sand was 32–36%, silt was 47–51% and clay was 12–20%, respectively.

In each of the six locations, 12 individual fields were chosen, and one soil profile in each field plot was dug out for the soil sampling with a 100-cm^3^ cutting ring. In total, 360 soil samples (6 locations × 12 field plots × 5 soil depths in the 1 m profile) were collected and placed in cloth bags, where they were air-dried until the weight was nearly constant. The 5 soil depths were respectively 0–20 cm, 20–40 cm, 40–60 cm, 60–80 cm and 80–100 cm. All roots and gravel were carefully removed, the soil samples were ground, and passed through a 0.25-mm sieve prior to laboratory analysis^50^.

### Extraction and determination of GRSP amount (EEG and TG)

Extraction and determination of GRSP amount in soil were conducted as described by Wright and Upadhyaya^70^. EEG was extracted from 0.5 g soil with 4 mL of 20 mmol L^−1^ citrates (pH 7.0) and autoclaved at 121 °C for 30 min. TG was extracted from 0.1 g soil with 4 mL of 50 mmol L^−1^ citrates (pH 8.0) and autoclaved at 121 °C for 1 h. In both cases, the solution products were separated by centrifugation at 6000 g for 6 min to collect the supernatant. For TG, the described procedure was repeated several times, combining all extract solution from the same soil sample, until the reddish-brown color, which is typical of GRSP disappeared from the supernatant. Protein amount in the crude extract was determined by the Bradford assay using bovine serum albumin as a standard^14,70^.

### Determination of soil properties

The SOC was measured by the Tinsley method (heated dichromate/ titration method). The total-N was determined by the Semimicro-Kjeldahl method. The amount of alkaline-hydrolyzable N (AN) was determined by the alkaline hydrolyzed diffusion method. Total P and K amounts were measured by the NaOH melt method. The available P (AP) was extracted with 0.05 N HCl–0.025 N H_2_SO_4_ solution and reacted with a solution of l-ascorbic acid and H_2_SO_4_–(NH_4_)_6_Mo_7_O_24_ methods. The available K (AK) was determined using flame photometry. All the above-mentioned methods were adapted from Bao^71^ and Wang, *et al*.^50^.

The pH of soil solution (1 g soil in 5 mL deionized water) was measured using a pH-meter (Sartorius PT-21; Shanghai, China). The same soil solution was used for determining soil EC using an EC meter (DDS-307; Shanghai Precision Scientific Instruments Co., Ltd., China). Both pH and EC measurement were determined at the same time. Soil gravimetric water was calculated as follows: [(Fresh weight − Air-dried weight) / Dry weight] × 100%. Soil bulk density was calculated as follows: Air-dried soil mass/soil volume of 400 cm^3^ ^50^.

### Data statistical analyses

The Multivariate Analysis of Variance (MANOVA) with Duncan pairwise comparison was used to compare the vertical variations in the glomalin features, soil physiochemical properties, and soil nutrient parameters. Pearson correlation, stepwise regression, Redundancy analysis (RDA) and structural equation model (SEM) analysis were used to uncover the associations between glomalin features and various factors of soil properties and climatic conditions, and possible differences between the surface and deep soils.

In the stepwise regression, criterion for the entering of a parameter is that inclusion at *p* < 0.01 and exclusion at *p* > 0.05; All the entered parameters for all the tested glomalin features were grouped as climatic conditions, soil physiochemical properties, and soil nutrients; The more parameters entered the stepwise models, the more important of these factors for regulating glomalin features.

The RDA-related variation partitioning was used to separate the explaining percentage into climatic conditions, soil physiochemical properties, soil nutrients and their interactions. The RDA ordination and variation partitioning were performed by Canoco 5.0 (Biometrics, Plant Research International, the Netherland).

SEM, as a supplement of traditional statistical analysis of linear regression and stepwise regression, allows for the specification of system-level network hypotheses. To clarify, traditional statistical models are of the form *y* = *f* (**X**), where y is some response variable of interest and **X** is a vector of predictor variables. However, this equational form provides no means for representing hypotheses about why *x* variables might be correlated. In contrast, SEMs are of the form **Y** = *f* (**X**, **Y**), which allows for the specification of network hypotheses in which each variable is seen to be part of a system of variables. As a result, we may test the idea that variable *C* is influenced by the variable *A* through the mediating effect of *B* (i.e., *A* → *B* → *C*). This flexibility in equational representation has numerous benefits, including the representation of complete hypotheses and the discovery of unanticipated relationships (e.g., effects of *A* on *C* not through *B*)^72^. In this paper, SEM was used in identifying the direct and indirect effects from soil physiochemical properties, soil nutrient parameters and climatic conditions on glomalin features (EEG, TG, EEG/TG, glomalin/SOC, glomalin/N, glomalin/P) in the surface soils (0–40 cm) and deep soils (40–100 cm) separately. The differences between the surface and deep soils were used to describe casual relations for the glomalin vertical pattern with soil properties and climatic differences. Too many independent factors will hinder the explaining of independent factors on dependent glomalin features, thus the PCA method was used to extract the main information from the data of climatic conditions, soil physiochemical properties and nutrient parameters for simplifying the complex association in the SEM analysis. The criterion for principal component selections is that eigenvalue larger than 1.0. The analysis was performed by IBM SPSS AMOS 22.0 (IBM, Armonk, NY) for the SEM analysis and SPSS 22.0 (IBM, Armonk, NY) for the PCA extraction.

In all the analysis, glomalin features were characterized as TG, EEG, EEG/TG (relative ratio of the EEG to TG), TG/SOC (relative ratio of the carbon in TG to total SOC), EEG/SOC(relative ratio of the carbon in EEG to total SOC), TG/N (relative ratio of the nitrogen in TG to total soil N), EEG/N (relative ratio of the nitrogen in EEG to total soil N), TG/P (relative ratio of the phosphorus in TG to total soil P) and EEG/P (relative ratio of the phosphorus in EEG to total soil P). The C (36.8%), N (3.7%), P (0.5%) and K (2.9%) content in extracted glomalin were cited from previous publication^14^ and used to compute the C, N, P and K amounts in EEG and TG. And these data were used in calculating glomalin contribution to SOC (EEG/SOC, TG/SOC), N (EEG/N, TG/N), and P (EEG/P, TG/P). The glomalin contribution to K is 0.26% for TG and 0.03% for EEG, and they are ignored in the analysis owing to their neglectable amount. For finding the differences between the surface and deep soils, the association analysis (Pearson correlation, stepwise regression, RDA and SEM) were separately analyzed in 0–40 cm soils (surface) and 40–100 cm soils (deep).

### Data statistical analyses

The datasets generated during and/or analysed during the current study are available from the corresponding author on reasonable request.

## Electronic supplementary material

Supplementary Information
