# Supplementary material for: Glomalin contributed more to carbon, nutrients in deeper soils, and differently associated with climates and soil properties in vertical profiles
Source: Sci Rep. 2017 Oct 11;7:13003. doi: 10.1038/s41598-017-12731-7 (PMC5636888; doi:10.1038/s41598-017-12731-7)
Supplement: Supplementary file 1 — Supplementary Information [file 41598_2017_12731_MOESM1_ESM.doc]

**Glomalin contributed more to carbon, nutrients in deeper soils, and differently associated with climates and soil properties in vertical profiles**

Wang Wenjie^1*^, Zhong Zhaoliang^1^, Wang Qiong^1,2^, Wang Huimei^1^,Fu Yujie^1^,He Xingyuan^2^

1: Key laboratory of Forest Plant Ecology, Northeast Forestry University, Harbin 150040, P.R. China

2. Urban forest and urban wetland group, Northeast Institute of Geography and Agroecology, CAS, Changchun 130102, P.R. China

*Correspondence author: wjwang225@hotmail.com; [wangwenjie@iga.ac.cn](mailto:wangwenjie@iga.ac.cn); wwj225@nefu.edu.cn

Table A1. Statistics of the principal analysis for climatic conditions, soil physiochemical properties and soil nutrients.

| Principal Component | Eigenvalue | |  | Selected sum load entered | | |
| --- | --- | --- | --- | --- | --- | --- |
|  | Sum | Variation percent % | Variation accumulation % | Sum | Variation percent % | Variation accumulation % |
| Climatic Component analysis | | |  |  |  |  |
| 1 | 2.2 | 73.6 | 73.6 | 2.2 | 73.6 | 73.6 |
| 2 | 0.5 | 17.8 | 91.4 |  |  |  |
| 3 | 0.3 | 8.6 | 100.0 |  |  |  |
| Soil physiochemical properties component analysis | | | | | |  |
| 1 | 1.4 | 35.7 | 35.7 | 1.4 | 35.7 | 35.7 |
| 2 | 1.1 | 28.2 | 63.9 | 1.1 | 28.2 | 63.9 |
| 3 | 0.8 | 19.6 | 83.5 |  |  |  |
| 4 | 0.7 | 16.5 | 100.0 |  |  |  |
| Soil nutrient component analysis | | | |  |  |  |
| 1 | 3.0 | 42.8 | 42.8 | 3.0 | 42.8 | 42.8 |
| 2 | 1.0 | 14.9 | 57.6 | 1.0 | 14.9 | 57.6 |
| 3 | 0.9 | 13.1 | 70.7 |  |  |  |
| 4 | 0.8 | 11.3 | 82.0 |  |  |  |
| 5 | 0.6 | 8.1 | 90.1 |  |  |  |
| 6 | 0.5 | 7.5 | 97.6 |  |  |  |
| 7 | 0.2 | 2.4 | 100.0 |  |  |  |

Table A2. Standard coefficient for the principal component analysis. Larger coefficient accompanies the larger contribution of this parameter to the principal component.

|  | PCA component 1 | PCA component 2 |
| --- | --- | --- |
| Climatic Component analysis | | |
| MAT | 0.36 |  |
| MAP | 0.39 |  |
| Altitude | 0.41 |  |
| Soil physiochemical properties component analysis | | |
| Bulkdensity | 0.51 | -0.24 |
| Soilwater | -0.56 | 0.06 |
| pH | 0.37 | 0.52 |
| EC | -0.05 | 0.74 |
| Soil nutrient component analysis | | |
| SOC | 0.30 | -0.01 |
| N | 0.29 | -0.08 |
| AN | 0.24 | 0.04 |
| AP | 0.09 | 0.62 |
| P | 0.24 | -0.04 |
| K | -0.09 | 0.72 |
| AK | 0.17 | 0.22 |

Table A3. Coefficients for the casual relations between soil depths, climatic conditions, soil nutrients and soil physiochemical properties on glomalin features from SEM analysis. S.E. is the standard error of the regression estimated. C.R.is the critical ratio for identifying the statistical significance of the regression weights.

| Effect factors | Direct | Casual factors | Regression weights | | | | Standard regression weights |
| --- | --- | --- | --- | --- | --- | --- | --- |
|  |  |  | Estimate | S.E. | C.R. | P | Estimate |
| NutrientPCA2 | <--- | depth | 0.005 | 0.002 | 2.703 | 0.007 | 0.141 |
| NutrientPCA1 | <--- | depth | -0.025 | 0.001 | -19.92 | *** | -0.698 |
| PhysiochemicalPCA2 | <--- | depth | -0.005 | 0.002 | -2.654 | 0.008 | -0.137 |
| PhysiochemicalPCA1 | <--- | depth | 0.011 | 0.002 | 7.227 | *** | 0.324 |
| NutrientPCA2 | <--- | ClimaticPCA1 | -0.033 | 0.052 | -0.624 | 0.533 | -0.033 |
| NutrientPCA1 | <--- | ClimaticPCA1 | 0.271 | 0.035 | 7.736 | *** | 0.271 |
| PhysiochemicalPCA2 | <--- | ClimaticPCA1 | -0.174 | 0.051 | -3.386 | *** | -0.174 |
| PhysiochemicalPCA1 | <--- | ClimaticPCA1 | -0.414 | 0.045 | -9.232 | *** | -0.414 |
| EEG | <--- | PhysiochemicalPCA1 | -0.086 | 0.014 | -6.207 | *** | -0.264 |
| EEG | <--- | PhysiochemicalPCA2 | -0.129 | 0.012 | -10.73 | *** | -0.398 |
| EEG | <--- | NutrientPCA1 | 0.106 | 0.018 | 5.987 | *** | 0.326 |
| EEG | <--- | NutrientPCA2 | 0.054 | 0.012 | 4.567 | *** | 0.167 |
| EEG | <--- | ClimaticPCA1 | -0.111 | 0.014 | -7.897 | *** | -0.342 |
| EEG | <--- | depth | -0.003 | 0.001 | -4.824 | *** | -0.264 |
| TG | <--- | PhysiochemicalPCA1 | -0.112 | 0.099 | -1.128 | 0.259 | -0.048 |
| TG | <--- | PhysiochemicalPCA2 | -0.455 | 0.086 | -5.264 | *** | -0.196 |
| TG | <--- | NutrientPCA1 | 1.282 | 0.127 | 10.10 | *** | 0.554 |
| TG | <--- | NutrientPCA2 | 0.259 | 0.085 | 3.046 | 0.002 | 0.112 |
| TG | <--- | ClimaticPCA1 | 0.305 | 0.101 | 3.022 | 0.003 | 0.132 |
| TG | <--- | depth | -0.01 | 0.005 | -2.315 | 0.021 | -0.127 |
| EEGSOC | <--- | PhysiochemicalPCA1 | 0.002 | 0.001 | 2.996 | 0.003 | 0.136 |
| EEGSOC | <--- | PhysiochemicalPCA2 | -0.002 | 0.001 | -3.802 | *** | -0.164 |
| EEGSOC | <--- | NutrientPCA1 | -0.006 | 0.001 | -7.586 | *** | -0.439 |
| EEGSOC | <--- | NutrientPCA2 | 0 | 0.001 | -0.221 | 0.825 | -0.008 |
| EEGSOC | <--- | ClimaticPCA1 | -0.002 | 0.001 | -2.963 | 0.003 | -0.141 |
| EEGSN | <--- | PhysiochemicalPCA1 | 0.003 | 0.001 | 4.468 | *** | 0.182 |
| EEGSN | <--- | PhysiochemicalPCA2 | -0.002 | 0.001 | -2.382 | 0.017 | -0.092 |
| EEGSN | <--- | NutrientPCA1 | -0.009 | 0.001 | -9.902 | *** | -0.515 |
| EEGSN | <--- | NutrientPCA2 | 0.001 | 0.001 | 1.397 | 0.162 | 0.048 |
| EEGSN | <--- | ClimaticPCA1 | -0.001 | 0.001 | -1.661 | 0.097 | -0.071 |
| EGTP | <--- | PhysiochemicalPCA1 | -0.001 | 0.005 | -0.313 | 0.755 | -0.02 |
| EGTP | <--- | PhysiochemicalPCA2 | -0.001 | 0.004 | -0.288 | 0.773 | -0.018 |
| EGTP | <--- | NutrientPCA1 | -0.005 | 0.006 | -0.757 | 0.449 | -0.062 |
| EGTP | <--- | NutrientPCA2 | -0.005 | 0.004 | -1.211 | 0.226 | -0.065 |
| EGTP | <--- | ClimaticPCA1 | -0.007 | 0.005 | -1.493 | 0.136 | -0.1 |
| EEGSOC | <--- | EEG | 0.029 | 0.002 | 11.85 | *** | 0.634 |
| EEGSN | <--- | EEG | 0.04 | 0.003 | 16.12 | *** | 0.773 |
| EGTP | <--- | EEG | 0.03 | 0.017 | 1.757 | 0.079 | 0.132 |
| EEGSOC | <--- | depth | 0 | 0 | 1.386 | 0.166 | 0.079 |
| EEGSN | <--- | depth | 0 | 0 | 0.587 | 0.557 | 0.03 |
| EGTP | <--- | depth | 0 | 0 | 1.848 | 0.065 | 0.149 |
| TGSOC | <--- | PhysiochemicalPCA1 | 0.013 | 0.004 | 3.397 | *** | 0.15 |
| TGSOC | <--- | PhysiochemicalPCA2 | -0.006 | 0.003 | -1.653 | 0.098 | -0.066 |
| TGSOC | <--- | NutrientPCA1 | -0.054 | 0.006 | -9.732 | *** | -0.625 |
| TGSOC | <--- | NutrientPCA2 | 0.004 | 0.003 | 1.222 | 0.222 | 0.047 |
| TGSOC | <--- | ClimaticPCA1 | 0.006 | 0.004 | 1.615 | 0.106 | 0.074 |
| TGSN | <--- | PhysiochemicalPCA1 | 0.01 | 0.004 | 2.476 | 0.013 | 0.1 |
| TGSN | <--- | PhysiochemicalPCA2 | -0.005 | 0.004 | -1.309 | 0.19 | -0.048 |
| TGSN | <--- | NutrientPCA1 | -0.074 | 0.006 | -12.65 | *** | -0.743 |
| TGSN | <--- | NutrientPCA2 | 0.011 | 0.003 | 3.135 | 0.002 | 0.11 |
| TGSN | <--- | ClimaticPCA1 | 0.002 | 0.004 | 0.408 | 0.683 | 0.017 |
| TGTP | <--- | PhysiochemicalPCA1 | -0.005 | 0.028 | -0.178 | 0.859 | -0.011 |
| TGTP | <--- | PhysiochemicalPCA2 | -0.004 | 0.025 | -0.142 | 0.887 | -0.008 |
| TGTP | <--- | NutrientPCA1 | -0.061 | 0.04 | -1.519 | 0.129 | -0.135 |
| TGTP | <--- | NutrientPCA2 | -0.036 | 0.024 | -1.497 | 0.134 | -0.08 |
| TGTP | <--- | ClimaticPCA1 | -0.044 | 0.029 | -1.536 | 0.125 | -0.097 |
| EGTG | <--- | PhysiochemicalPCA1 | -0.005 | 0.012 | -0.468 | 0.64 | -0.023 |
| EGTG | <--- | PhysiochemicalPCA2 | -0.022 | 0.011 | -1.914 | 0.056 | -0.091 |
| EGTG | <--- | NutrientPCA1 | 0.03 | 0.017 | 1.796 | 0.072 | 0.125 |
| EGTG | <--- | NutrientPCA2 | -0.01 | 0.01 | -0.996 | 0.319 | -0.041 |
| EGTG | <--- | ClimaticPCA1 | -0.021 | 0.012 | -1.667 | 0.096 | -0.086 |
| EGTG | <--- | depth | 0 | 0.001 | 0.692 | 0.489 | 0.043 |
| TGTP | <--- | depth | 0.002 | 0.001 | 1.686 | 0.092 | 0.134 |
| TGSOC | <--- | depth | 0.001 | 0 | 2.93 | 0.003 | 0.168 |
| TGSN | <--- | depth | 0 | 0 | 1.045 | 0.296 | 0.055 |
| EGTG | <--- | EEG | 0.315 | 0.042 | 7.488 | *** | 0.43 |
| EGTG | <--- | TG | -0.074 | 0.006 | -12.44 | *** | -0.716 |
| TGSOC | <--- | TG | 0.031 | 0.002 | 15.26 | *** | 0.839 |
| TGSN | <--- | TG | 0.041 | 0.002 | 19.07 | *** | 0.957 |
| TGTP | <--- | TG | 0.041 | 0.015 | 2.791 | 0.005 | 0.213 |
